# Formulation and Optimization of a *Melissa officinalis*-Loaded Nanoemulgel for Anti-Inflammatory Therapy Using Design of Experiments (DoE)

**DOI:** 10.3390/gels11100776

**Published:** 2025-09-26

**Authors:** Yetukuri Koushik, Nadendla Rama Rao, Uriti Sri Venkatesh, Gottam Venkata Rami Reddy, Amareswarapu V. Surendra, Thalla Sreenu

**Affiliations:** 1 Department of Pharmaceutics, Chalapathi Institute of Pharmaceutical Sciences, Lam, Guntur 522034, Andhra Pradesh, India; nadendla2000@yahoo.co.in (N.R.R.); ramreddyv444@gmail.com (G.V.R.R.); 2 Department of Pharmacology, Sri Sivani College of Pharmacy, Chilakapalem Junction, Etcherla, Srikakulam 532410, Andhra Pradesh, India; venkateshbalaji230@gmail.com; 3 Department of Pharmaceutics, KL College of Pharmacy, Koneru Lakshmaiah Education Foundation, Green Fields, Vaddeswaram, Guntur 522502, Andhra Pradesh, India; buddi0707@gmail.com; 4 Department of Pharmacology, Vignan Pharmacy College, Vadlamudi, Guntur 522213, Andhra Pradesh, India; sreenuthalla87@gmail.com

**Keywords:** nanoemulsion, *Melissa officinalis*, transdermal system, anti-inflammatory, antimicrobial, controlled drug release, Design of Experiments

## Abstract

This study reports the development and optimization of a *Melissa officinalis* oil-based nanoemulgel for transdermal delivery using a Design-of-Experiments (DoE) approach. A Central Composite Design (CCD) was applied to optimize Tween 80 concentration and homogenization time, resulting in a nanoemulsion with a droplet size of 127.31 nm, PDI of 17.7%, and zeta potential of −25.0 mV, indicating good colloidal stability. FTIR analysis confirmed the presence of functional groups such as O–H, C=O, and C–O–C, supporting the oil’s phytochemical richness and therapeutic potential. DSC analysis revealed enhanced thermal stability and successful encapsulation, while SEM imaging showed a uniform and spherical microstructure. The drug release followed Higuchi kinetics (R^2^ = 0.900), indicating diffusion-driven release, with the Korsmeyer–Peppas model (*n* = 0.88) suggesting anomalous transport. Antibacterial studies showed inhibition of *Staphylococcus aureus* (MIC = 250 µg/mL) and *Escherichia coli* (MIC = 500 µg/mL). In vivo anti-inflammatory testing demonstrated significant edema reduction (*p* < 0.05) using a carrageenan-induced rat paw model. These results support the potential of Melissa nanoemulgel as a stable and effective topical therapeutic for inflammatory and microbial skin disorders.

## 1. Introduction

Human skin, the body’s largest organ, performs diverse roles by forming a dynamic boundary between internal physiology and the external surroundings. It acts as a crucial physical, chemical, and immunological defense mechanism. Anatomically, the skin consists of three primary strata—epidermis, dermis, and hypodermis—each of which contributes to maintaining internal equilibrium, defending against microbial invasion, and initiating wound repair mechanisms. However, the skin often faces damage from external insults such as UV radiation, pollutants, and pathogenic microorganisms, alongside intrinsic contributors like aging and persistent inflammation. These factors collectively lead to increased reactive oxygen species (ROS) production, immune system disturbances, and compromise of the skin’s protective function, ultimately playing a role in the onset of inflammatory skin conditions such as eczema and psoriasis [1,2]. In recent years, essential oils (EOs), particularly those derived from medicinal plants, have gained significant attention due to their broad-spectrum pharmacological properties, including antimicrobial, antioxidant, and anti-inflammatory activities. These therapeutic effects are attributed to their complex composition of bioactive molecules such as terpenoids, aldehydes, and phenolic compounds. *Melissa officinalis* (lemon balm) essential oil, rich in constituents like citral, citronellal, and rosmarinic acid, has demonstrated potential in mitigating inflammatory responses through modulation of oxidative stress and suppression of pro-inflammatory cytokines such as TNF-α and IL-6 [3]. Owing to these properties, *Melissa officinalis* is increasingly being investigated for managing inflammatory skin conditions.

Nevertheless, the clinical utility of essential oils in topical therapy is limited by their inherent volatility, poor aqueous solubility, and low permeability across the stratum corneum. Nanocarrier-based delivery systems have been extensively explored to overcome these challenges, offering improved stability, enhanced permeation, and better therapeutic performance. Among them, nanoemulsions, liposomes, solid lipid nanoparticles, and nanostructured lipid carriers have shown considerable promise [4,5]. Incorporating nanoemulsions into a gel matrix leads to the development of nanoemulgels—hybrid delivery systems that offer the dual benefits of nanoemulsion-based solubilization and gel-based retention. Nanoemulgels are thermodynamically stable, easily scalable, and facilitate prolonged drug residence time at the application site, which is particularly beneficial for localized treatment of inflammation [6,7]. Low-energy emulsification techniques used in their preparation are cost-effective and suitable for thermolabile components such as essential oils. The growing application of nanoemulgels and nanogels in inflammation management has been well-supported by recent studies. Enzyme-responsive nanogels composed of polyethylene glycol have demonstrated the controlled release of paraoxonase-1 (PON-1), resulting in a reduction in LDL oxidation and inflammation [8]. Similarly, sprayable zwitterionic chitosan nanogels synthesized via a green, organic solvent-free approach have shown multifunctional properties, including ROS scavenging, antibacterial effects, and anti-inflammatory activity [9]. Moreover, nanoemulsion-based gels incorporating cinnamon and clove essential oils have exhibited significant anti-inflammatory and anti-nociceptive effects in vivo, further validating the effectiveness of essential oil-loaded nanocarriers [10]. These advances establish a strong rationale for the development of *Melissa officinalis*-loaded nanoemulgels as an effective transdermal delivery system for anti-inflammatory therapy. In the present study, a *Melissa officinalis* nanoemulgel (M-NG) was formulated and optimized using a Central Composite Design (CCD) within the Quality by Design (QbD) framework to assess the effects of formulation parameters on critical quality attributes such as droplet size, zeta potential, and polydispersity index. The optimized formulation was characterized and further evaluated for its in vitro drug release, antimicrobial potential, and in vivo anti-inflammatory activity using a carrageenan-induced rat paw edema model. This integrated formulation and evaluation strategy aims to enhance the therapeutic efficacy and stability of essential oil-based topical systems for managing inflammatory skin conditions.

## 2. Results and Discussion

### 2.1. FT-IR Analysis of Melissa Oil

**Figure 1:**
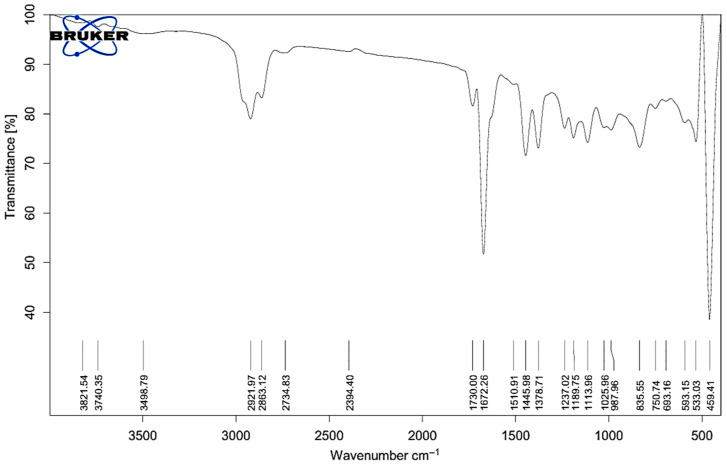
FT-IR spectrum of Melissa oil showing characteristic peaks for functional groups including O–H (3821–3498 cm^−1^), C–H (2921–2734 cm^−1^), C=O (1730, 1672 cm^−1^), C=C and CH bending (1510–1378 cm^−1^), and C–O (1237–1025 cm^−1^), confirming the presence of terpenes, esters, alcohols, and phenolic compounds.

The infrared spectrum of Melissa oil shows multiple functional moieties typical of essential oils, underscoring its rich phytochemical content. Spectra were acquired on a Bruker ALPHA II instrument fitted with an ATR module. Measurements covered 4000–400 cm^−1^ at 4 cm^−1^ resolution, with 32 co-added scans to enhance signal reliability (Figure 1). A broad, intense band at 3821.54–3740.35 cm^−1^ corresponds to O–H stretching of free hydroxyl groups, indicative of alcohols and phenolics. A feature at 3498.79 cm^−1^ suggests hydrogen-bonded O–H vibrations. Pronounced C–H stretching absorptions appear at 2921.97, 2863.12, and 2734.83 cm^−1^, confirming aliphatic chains—particularly methyl and methylene groups—in the oil.

A minor peak at 2394.40 cm^−1^ might be attributed to overtones or trace compounds. Strong absorption bands at 1730.00 and 1672.26 cm^−1^ are assigned to C=O stretching, suggesting the presence of esters and ketones, which are common bioactive components in Melissa oil. Peaks at 1510.91, 1445.89, and 1378.71 cm^−1^ further support the presence of C=C (aromatic or alkene) and C–H bending vibrations typical of alkane chains. The C–O stretching region showed significant peaks at 1237.02, 1180.75, 1113.96, and 1025.96 cm^−1^, indicating ether or alcohol functionalities, while peaks at 987.69 and 925.59 cm^−1^ could be associated with out-of-plane bending of =C–H groups or cyclic terpenes. Distinct absorption in the fingerprint region includes peaks at 835.55, 750.74, 693.16, 593.15, 533.13, and 459.41 cm^−1^, contributing to the unique identity of Melissa oil. These spectral features confirm the presence of key chemical constituents such as terpenes, esters, alcohols, and phenolics. From a formulation perspective, hydroxyl (–OH) and carbonyl (C=O) groups may enhance emulsification and solubility due to their polar nature, thereby improving nanoemulsion stability. The C–O–C ether linkages and phenolic content also contribute to the antioxidant and antimicrobial properties of the oil, which can help protect the formulation from oxidative degradation and microbial contamination. In addition, monoterpenes and sesquiterpenes—known for their membrane-permeating and anti-inflammatory activities—support the therapeutic effectiveness of the nanoemulgel in transdermal applications. These observations confirm the presence of essential oil constituents that reinforce both the chemical stability and biological performance of the formulation. The functional groups identified from the recorded absorption bands are detailed in Table 1, which lists the standard wavenumber intervals associated with each group. These spectral results affirm the existence of bioactive constituents such as monoterpenes, sesquiterpenes, esters, alcohols, and phenolic derivatives, thereby confirming the phytochemical composition of Melissa oil and reinforcing its suitability for use in both therapeutic and cosmeceutical product development.

### 2.2. Preparation and Optimization of MelissaNanoemulsions

*Melissa*–oil nanoemulsions were optimized through a statistical response surface design, with droplet diameter and zeta potential serving as key quality parameters. The experimental matrix listed both real and coded settings for two process factors: the surfactant level (Tween 80, X_1_) and the duration of homogenization (X_2_), selected due to their strong influence on emulsification efficiency and colloidal stability (Table 2). The objective was to obtain the smallest possible droplets while preserving electrokinetic stability. Results showed that both factors significantly affected droplet size. The minimum diameter (110.98 nm) occurred when Tween 80 was at its midpoint (X_1_ = 0) and homogenization time was at the upper axial value (X_2_ = A, 34.14 min), implying that prolonged high-shear processing narrows the size distribution. Conversely, the largest droplets (201.1 nm) formed when both variables were at their lowest settings (X_1_ = −, X_2_ = −), reflecting inadequate surfactant content and mechanical energy for effective emulsification.

Excess Tween 80 (X_1_ = A or +) did not yield substantial improvements in droplet size reduction, implying the presence of a saturation point beyond which emulsification efficiency plateaus. Similarly, higher homogenization times continued to benefit particle size reduction up to a certain threshold. The optimal formulation was characterized by moderate Tween 80 (4%) and extended homogenization (34.14 min), striking a balance between emulsifier availability and mechanical energy. Following regression analysis using Design Expert (Version 13) software, a polynomial model incorporating squared terms was generated to describe the influence of formulation factors on particle size:

PZ = 143.92 − 23.56X_1_ − 27.89X_2_ + 1.89X_1_X_2_ + 9.63X_1_^2^ + 5.74X_2_^2^

where *PZ* represents particle size (nm), *X*_1_ denotes Tween 80 concentration (coded values for 2–6%), and *X*_2_ refers to homogenization time (coded values for 10–30 min). The model indicates that both Tween 80 and homogenization time exert a significant negative linear effect on particle size, contributing to size reduction. However, the positive quadratic terms suggest that beyond certain optimal points, further increases may not enhance the formulation performance. The model’s statistical validity was supported by ANOVA (Table 3), with an overall *p*-value of 0.0021, indicating high model significance. Individual terms for Tween 80 (*p* = 0.0047) and homogenization time (*p* = 0.0022) were also highly significant. The quadratic term for Tween 80 (*p* = 0.0481) was marginally significant, suggesting a non-linear influence. In contrast, the interaction between Tween 80 and homogenization time was not significant (*p* = 0.7442), indicating that the two factors function independently in their effects on particle size.

The model exhibited a coefficient of determination (R^2^) of 0.8992, demonstrating that 89.92% of the variability in particle size can be explained by the model. The adjusted R^2^ of 0.8465 confirms the model’s reliability after considering the number of predictors.

**Figure 2:**
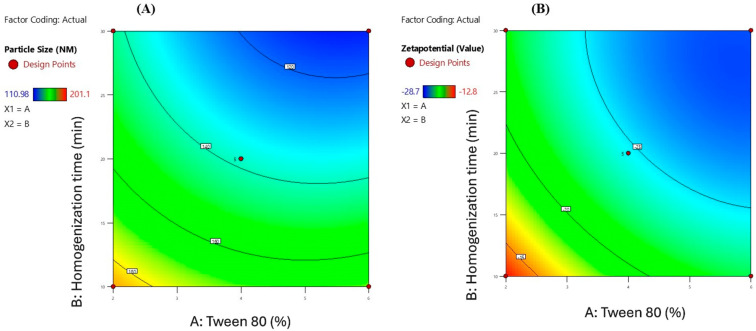
Contour plots depicting the interactive impact of Tween 80 (%) and homogenization time (minutes) on (**A**) particle size (nm) and (**B**) zeta potential (mV) of Melissa oil nanoemulsions. The plots demonstrate that increasing Tween 80 concentration and homogenization time significantly reduces particle size and enhances the negative surface charge, indicating improved nanoemulsion stability.

The contour plots illustrate the combined influence of Tween 80 percentage (X_1_) and homogenization time (X_2_) on the physicochemical characteristics of Melissa oil nanoemulsions, specifically particle size and zeta potential (Figure 2). The particle size contour plot (A) reveals a pronounced decrease in particle size with increasing Tween 80 concentration from 2% to 6% and prolonged homogenization time from 10 to 30 min, achieving a minimum size near 110.98 nm. This reduction is attributed to enhanced surfactant-mediated interfacial tension reduction and increased mechanical shear, facilitating effective droplet disruption and stabilization. The zeta potential contour plot (B) demonstrates that higher Tween 80 concentrations and extended homogenization times result in increasingly negative zeta potential values, reaching approximately −28.7 mV. The elevated negative surface charge promotes electrostatic repulsion between dispersed droplets, thereby enhancing colloidal stability by preventing coalescence and aggregation. Collectively, these contour plots substantiate that optimizing both surfactant concentration and homogenization duration is critical for producing stable Melissa oil nanoemulsions with reduced particle size and enhanced surface charge, which are essential parameters for improved formulation performance and shelf-life stability.

**Figure 3:**
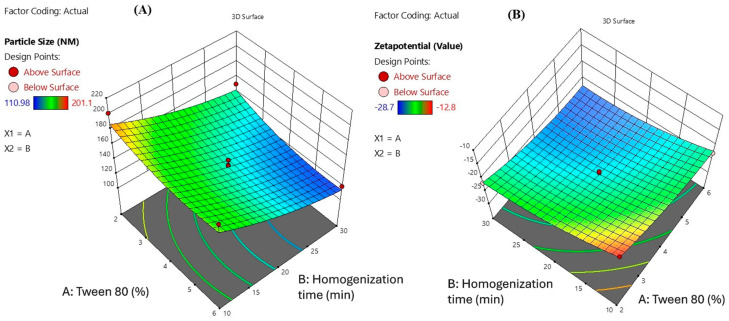
Three-dimensional surface plots illustrating the combined effects of Tween 80 concentration (X_1_) and homogenization time (X_2_) on (**A**) particle size and (**B**) zeta potential of Melissa oil nanoemulsion. The plots demonstrate that increasing Tween 80 concentration and homogenization time leads to a significant decrease in particle size and a more negative zeta potential, indicating enhanced emulsification efficiency and improved nanoemulsion stability.

The 3D surface plots presented illustrate the influence of Tween 80 concentration (X_1_) and homogenization time (X_2_) on the particle size and zeta potential of Melissa oil nanoemulsion (Figure 3). In the first plot (A), particle size is significantly affected by both variables. As the concentration of Tween 80 increases from 2% to 6% and the homogenization time extends from 10 to 30 min, a reduction in particle size is observed. The smallest particle size achieved is around 110.98 nm, indicating improved emulsification efficiency at higher surfactant levels and prolonged homogenization, likely due to enhanced shear force and better droplet breakup. In the second plot (B), zeta potential values become more negative with increasing Tween 80 concentration and homogenization time, reaching a value of 28.7 mV. This trend signifies enhanced physical stability of the nanoemulsion, as higher negative zeta potential values indicate stronger electrostatic repulsion between droplets, preventing aggregation. Overall, these plots confirm that optimal formulation parameters higher Tween 80 concentration and adequate homogenization time are critical for achieving stable nanoemulsions with small particle sizes and improved surface charge stability.

**Figure 4:**
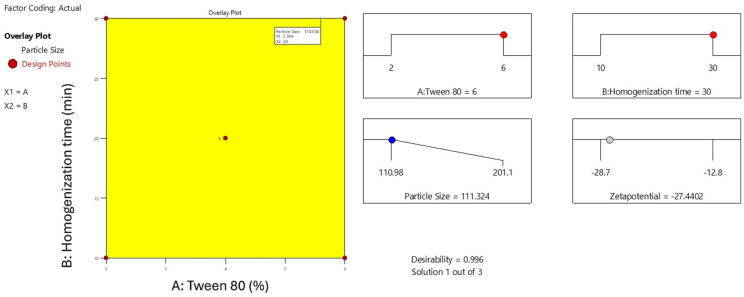
Overlay plot depicting the optimal design space for particle size reduction in Melissa oil nanoemulsion. The yellow region indicates the interaction between Tween 80 concentration (%) and homogenization time (minutes) that results in minimized particle size (114 nm). Red dots represent experimental design points, with the central point marking the optimum formulation conditions.

The overlay plot illustrates the optimized formulation space for minimizing particle size in the Melissa oil nanoemulsion by adjusting Tween 80 concentration (X_1_) and homogenization time (X_2_) (Figure 4). The highlighted yellow area represents the design space where the particle size meets the desired minimum criteria, approximately 114 nm. Red points mark the experimental design runs, with the central point indicating the optimal condition of 5.6% Tween 80 and 30 min homogenization time. This plot emphasizes the synergistic effect of surfactant concentration and homogenization duration on reducing particle size, which is critical for achieving enhanced stability and efficacy of the nanoemulsion. The overlay visualization aids in clearly defining the optimal parameters for formulation development.

### 2.3. Characterization Study of Melissa-Loaded Nanoemulsions (M-NE)

To identify a formulation with optimal performance, freshly developed Melissa oil nanoemulsions (M-NEs) were subjected to a series of characterization and stability assessments. Over a 60-day observation period under varying thermal storage conditions, the formulations demonstrated excellent physical stability, with no visible signs of creaming, phase separation, or noticeable alterations in color or transparency. Furthermore, thermodynamic stability was verified through successive heating-cooling and freeze–thaw cycles, indicating the formulation’s resilience against temperature-induced stress. Based on the design space predicted through CCD and the associated prediction profiles, the most stable formulation exhibiting the most favorable physicochemical attributes was selected for subsequent investigations.

### 2.4. Physicochemical Characteristics and Morphological Analysis

**Figure 5:**
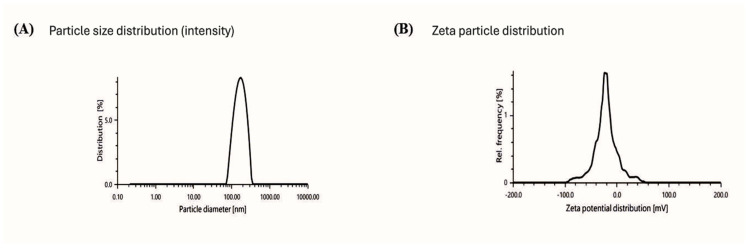
(**A**) Particle size distribution of Melissa nanoemulsion showing a uniform average diameter of 127.31 nm with a polydispersity index (PDI) of 17.7%, indicating good homogeneity. (**B**) Zeta potential distribution exhibited a value of −25.0 mV, suggesting adequate electrostatic repulsion and colloidal stability.

The particle size analysis of the Melissa oil nanoemulsion revealed a hydrodynamic diameter of 127.31 nm with a polydispersity index (PDI) of 17.7%, indicating a moderately uniform distribution and stable dispersion. The transmittance value of 88.3% further supports the clarity and homogeneity of the formulation. Zeta potential measurements exhibited a value of −25.0 mV, suggesting good colloidal stability by ensuring sufficient electrostatic repulsion between particles, thereby preventing aggregation. The standard deviation of 0.7 mV and a distribution peak at −23.5 mV confirm the consistency of the surface charge across the sample. Overall, these findings indicate that the Melissa oil nanoemulsion possesses suitable physicochemical properties for potential transdermal or topical application, offering stable droplet distribution and robust colloidal stability (Figure 5).

### 2.5. Scanning Electron Microscopy (SEM) Assessment

**Figure 6:**
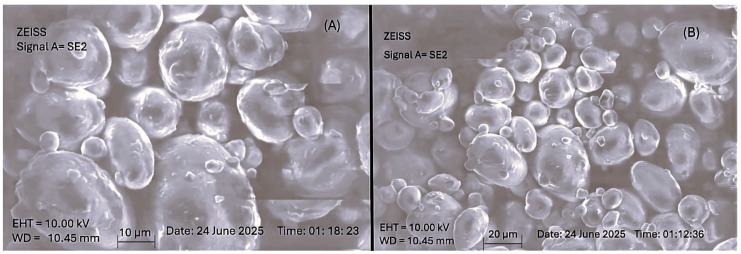
Scanning Electron Microscopy (SEM) images of the optimized Melissa nanoemulsion formulation. (**A**) SEM micrograph with a 10 μm scale bar reveals spherical droplets with smooth surfaces and uniform morphology. (**B**) SEM micrograph with a 20 μm scale bar demonstrates homogenous distribution of nanoemulsion particles without significant aggregation, confirming good physical stability of the formulation.

The SEM micrographs of the Melissa nanoemulsion (Figure 6) displayed a uniform, spherical particle morphology, characteristic of successfully formulated oil-in-water nanoemulsions. The droplets appeared well-defined with smooth, non-porous surfaces, suggesting effective stabilization of the oil phase by Tween 80. The uniformity in size and shape distribution across the field of view indicates minimal coalescence or aggregation, which supports the dynamic light scattering (DLS) data showing a narrow size distribution and a low polydispersity index (PDI). Additionally, the spherical appearance with no collapsed or ruptured structures indicates that the lyophilization process preserved the internal architecture of the nanoemulsion droplets. This is indicative of a well-optimized formulation and robust surfactant interface. The absence of surface cracks or deformities also points to good mechanical stability of the formulation matrix under processing stress. These morphological observations reinforce the earlier physicochemical characterizations such as particle size analysis and zeta potential, confirming that the developed nanoemulsion possesses suitable characteristics for further incorporation into gel matrices for transdermal delivery applications. The observed structural integrity and spherical shape are vital for achieving predictable release kinetics, dermal permeation, and therapeutic efficacy.

### 2.6. Entrapment Efficiency of Melissa Nanoemulsion

The optimized Melissa nanoemulsion (M-NE) formulation exhibited a high entrapment efficiency of 95.63 ± 0.47%, indicating effective encapsulation of Melissa oil within the nanoemulsion system. The entrapment efficiency was determined by separating the unentrapped oil through ultrafiltration, followed by spectrophotometric quantification at 274 nm. The total amount of oil added and the amount of free oil measured in the filtrate were used to calculate the percentage of encapsulated oil. A calibration curve prepared in the concentration range of 1–15 µg/mL showed strong linearity with a correlation coefficient (R^2^) of 0.998. The limit of detection (LOD) and limit of quantification (LOQ) were calculated as 0.46 µg/mL and 1.44 µg/mL, respectively, confirming the sensitivity and reliability of the method. On the day of preparation, the Melissa oil content in the formulation was 97.85 ± 0.38%. After 60 days of storage under controlled conditions, the content decreased to 91.72 ± 1.22%, corresponding to a retention rate of 93.74%. These findings confirm the long-term stability of the nanoemulsion system and support the robustness of the formulation process. The high entrapment is attributed to the effective emulsification by Tween 80 and the optimized homogenization conditions, which reduced diffusion of oil from the dispersed phase, ensuring efficient entrapment.

### 2.7. Design and Evaluation of a Melissa Nanoemulgel (M-NG)

The optimized *Melissa* nanoemulsion (M-NE) was effectively integrated into a hydrogel base to create Melissa nanoemulgel (M-NG), aiming to improve the formulation’s suitability for topical use. The base hydrogel was prepared with polyacrylic acid (Carbopol 940), dispersed in distilled water under continuous mixing. The mixture was then neutralized with triethanolamine to achieve a clear gel. Of the tested formulations, the hydrogel containing 1% *w*/*w* polyacrylic acid showed ideal physicochemical characteristics such as uniformity, transparency, stable appearance, and good adhesive qualities, as confirmed through the Thumb Test over a 30-day period. Thermal stability evaluation under accelerated storage conditions indicated that the M-NG retained structural integrity without visible evidence of separation, layering, or water release. Formulations with higher polymer concentrations resulted in increased viscosity and enhanced mechanical strength, which aided in maintaining stability during centrifugation; however, they exhibited somewhat lower spreadability, which may influence how easily they can be applied. The optimized M-NG formulation displayed a stable pH of 5.68 ± 0.07 over 30 days of storage, confirming skin compatibility and minimal potential for irritation. Assessments of appearance, consistency, and ease of application using the glass plate method and visual checks validated the consistency and strength of the formulation. These results highlight the promise of the *Melissa* nanoemulgel as a reliable and efficient carrier for skin-based therapeutic use.

### 2.8. Physicochemical Characterization of Nanoemulgel

**Figure 7:**
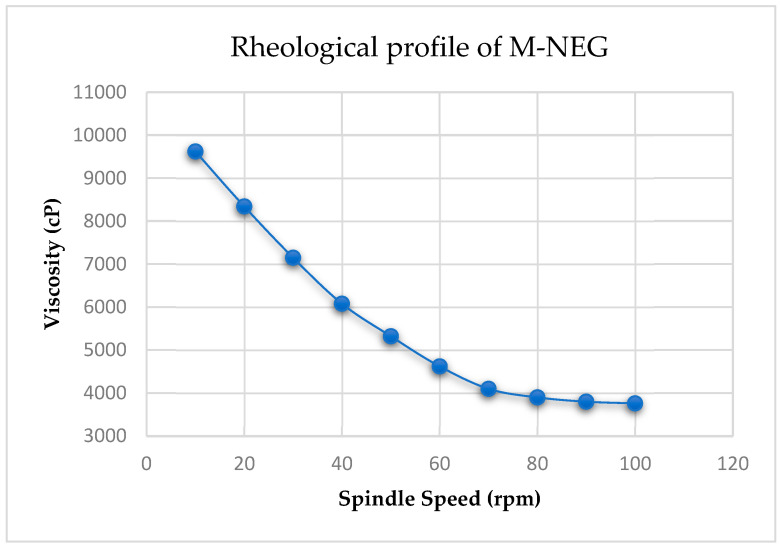
Rheological profile of Melissa nanoemulgel showing viscosity vs. spindle speed with error bars (±SD, *n* = 3). The decreasing trend confirms pseudoplastic behavior. Caps on error bars represent standard deviation limits.

The Melissa nanoemulgel was pale yellow, translucent, and homogenous in appearance, with no signs of phase separation or syneresis observed during a 7-day stability evaluation. The texture was smooth and non-greasy, spreading uniformly between glass slides without grittiness, indicating good physical stability and aesthetic acceptability. Rheological analysis confirmed pseudoplastic (shear-thinning) behavior, as evidenced by a progressive decrease in viscosity with increasing spindle speed. At 10 rpm, the viscosity was 9620 ± 120 cP, which decreased to 8340 ± 110 cP at 20 rpm, 7150 ± 105 cP at 30 rpm, and 6080 ± 95 cP at 40 rpm. Further reductions were observed at 50 rpm (5320 ± 90 cP), 60 rpm (4620 ± 85 cP), 70 rpm (4100 ± 82 cP), 80 rpm (3900 ± 78 cP), 90 rpm (3800 ± 76 cP), and finally 3760 ± 74 cP at 100 rpm. This consistent decline in viscosity with increasing shear indicates the shear-thinning nature of the formulation, which enhances spreadability during application while maintaining viscosity upon rest. These results are graphically presented in Figure 7, which includes error bars (±SD, *n* = 3) and capped ends to visually represent replicate variability. Spreadability testing showed an average spread diameter of 6.8 ± 0.2 cm under a 500 g load, indicating the formulation’s adequate extensibility for uniform application to the skin.

### 2.9. DSC Thermogram Analysis

Differential Scanning Calorimetry (DSC) analysis was conducted to evaluate the thermal behavior and physical state of *Melissa officinalis* (lemon balm) oil and the developed Melissa-loaded nanoemulgel. This analysis provides insight into the encapsulation efficiency, stability, and molecular interaction of the essential oil with the gel matrix, which is critical for ensuring performance in anti-inflammatory topical delivery.

**Figure 8:**
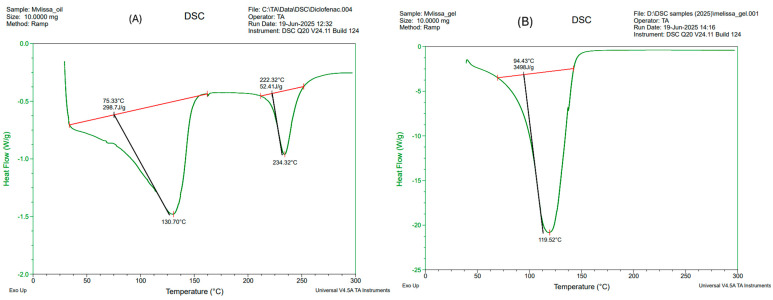
DSC thermograms of *Melissa officinalis* oil (**A**) and Melissa-loaded nanoemulgel formulation (**B**). The oil shows two thermal transitions representing evaporation of volatile and stable components, whereas the nanoemulgel exhibits a single broad transition indicating successful encapsulation and thermal stabilization of the oil within the gel matrix.

The DSC thermogram of pure Melissa oil (Figure 8A, left panel) revealed two distinct endothermic transitions, indicating its multicomponent nature. The first major endothermic peak appeared with an onset at 75.33 °C and a minimum at 130.70 °C, accompanied by an enthalpy change of 298.7 J/g. This thermal event is attributed to the volatilization or phase transition of low-boiling volatile constituents such as citral, neral, geranial, and limonene, which are known for their anti-inflammatory and antioxidant properties. A second, less intense peak was observed at 222.32 °C, ending around 234.32 °C, with an enthalpy change of 52.41 J/g, likely corresponding to the evaporation or decomposition of more thermally stable terpenes, including geraniol and sesquiterpenes. These transitions confirm the volatile and thermolabile nature of the oil, which presents formulation challenges in terms of maintaining bioactivity during processing and storage.

In contrast, the DSC thermogram of the Melissa-loaded nanoemulgel formulation (Figure 8B, right panel) exhibited a single broad endothermic event, beginning at 94.43 °C and peaking at 119.52 °C, with a significantly higher enthalpy of 349.8 J/g. The absence of multiple peaks and the shift in onset temperature to a higher range are indicative of a successful encapsulation of the essential oil within the gel network. The increase in enthalpy suggests stronger molecular interactions between the essential oil components and the gel excipients, possibly due to interfacial adsorption at the oil-surfactant boundary, physical entrapment within the polymeric matrix, or hydrogen bonding. This thermal stabilization is critical in preventing premature volatilization of active compounds, thereby enhancing the shelf-life and performance of the formulation upon topical application.

These results support the DoE optimized formulation strategy, as the thermal behavior confirms the compatibility and stability of the selected excipients with the active oil. The DSC findings thus corroborate the formulation’s suitability for sustained anti-inflammatory activity, validating its potential for transdermal drug delivery applications.

### 2.10. In Vitro Evaluation of Drug Release and Diffusion Profiles

**Figure 9:**
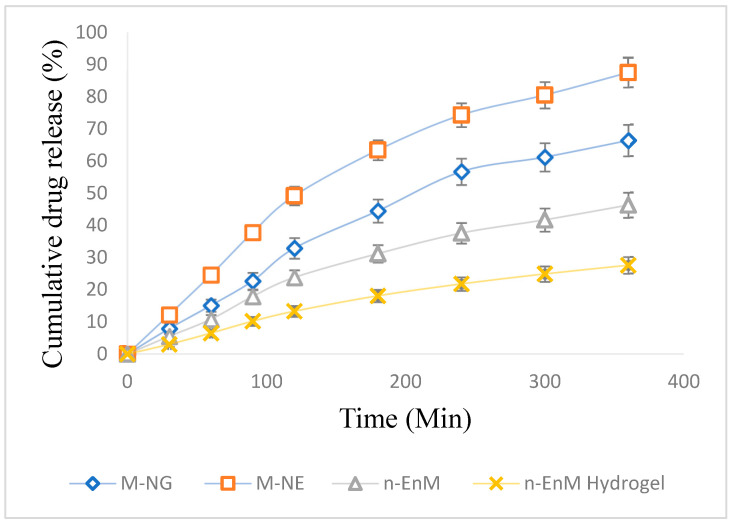
Percentage of cumulative drug release from various Melissa oil formulations over time, expressed as mean ± standard deviation (*n* = 3). The release curves for M-NG, M-NE, n-EnM, and n-EnM Hydrogel illustrate variations in drug release kinetics between the different formulations. Error bars represent standard deviation, indicating the extent of variability in the release patterns.

The rate and extent of drug release are vital parameters in assessing the effectiveness of topical formulations. To examine the release profile of Melissa oil from different formulations, a dialysis membrane method was employed over a period of 6 h (360 min). The study compared four distinct formulations: Melissa Nanoemulgel (M-NG), Melissa Nanoemulsion (M-NE), Melissa Nanoemulsion in Carbopol hydrogel base (n-EnM), and Melissa-loaded Hydrogel (n-EnM Hydrogel). The cumulative drug release (%) was measured at intervals of 30 min, and the mean values with standard deviations were recorded (Figure 9). At 30 min, M-NG demonstrated a drug release of 7.9 ± 1.1%, while M-NE showed 12.2 ± 1.6%, n-EnM released 5.5 ± 1.2%, and n-EnM Hydrogel had a release of 3.0 ± 0.7%. As time progressed, all formulations exhibited sustained release profiles. After 180 min, the drug release from M-NG reached 44.5 ± 3.1%, while M-NE released 63.4 ± 3.6%, n-EnM showed 31.2 ± 2.7%, and n-EnM Hydrogel recorded 18.1 ± 1.9%. A gradual increase was observed until the final time point at 360 min, where the cumulative release for M-NG was 66.4 ± 4.6%, slightly lower than M-NE (87.5 ± 4.9%), but higher than n-EnM (46.3 ± 3.9%) and n-EnM Hydrogel (27.6 ± 2.6%). These findings highlight the superior release potential of the nanoemulsion system, with the hydrogel matrix further modulating the release pattern. Among the tested systems, M-NE exhibited the highest release percentage, likely due to its smaller droplet size and absence of a gel matrix that might hinder diffusion. However, M-NG presented a more controlled and steady release, suggesting its suitability for sustained therapeutic action through topical delivery.

To further understand the mechanism of drug release from the M-NG formulation, the release data were fitted into various kinetic models, including zero-order, first-order, Higuchi, Korsmeyer–Peppas, and Hixson–Crowell. The correlation coefficient (R^2^) values for each model are summarized in Table 4. The Higuchi model showed the best fit (R^2^ = 0.900), indicating that the drug release was primarily diffusion-controlled. The Korsmeyer–Peppas model (R^2^ = 0.895) further supported an anomalous (non-Fickian) transport mechanism with a release exponent (*n*) of 0.88, suggesting that both diffusion and polymer relaxation contributed to drug release.

### 2.11. Accelerated Stability Evaluation

The Melissa nanoemulgel demonstrated excellent physical stability over the 30-day accelerated stability testing period. Throughout storage at 40 ± 2 °C and 75 ± 5% RH, the formulation retained its original pale-yellow color, semi-transparent appearance, and smooth, uniform consistency. No signs of phase separation, precipitation, or syneresis were observed upon visual inspection or after centrifugation, indicating good emulsion stability. Viscosity measurements showed only minor variation over time, with values recorded as 9620 ± 120 cP on Day 0, 9450 ± 135 cP on Day 15, and 9390 ± 142 cP on Day 30, confirming that the formulation maintained its rheological integrity under stress. Similarly, the average spread diameter changed insignificantly, from 6.8 ± 0.2 cm (Day 0) to 6.7 ± 0.2 cm (Day 30), suggesting preserved spreadability and application performance.

These results affirm that the M-NG formulation is physically stable for at least 30 days under accelerated storage conditions, which is a promising indicator for its long-term shelf-life and clinical usability pending further real-time studies.

### 2.12. Assessment of Antibacterial Efficacy

The antibacterial efficacy of the Melissa Nanoemulgel (M-NG) was evaluated against four bacterial strains using the broth dilution technique to determine the minimum inhibitory concentration (MIC). The results revealed that *Staphylococcus aureus*, a Gram-positive bacterium, was the most sensitive to M-NG with an MIC of 250 µg/mL, followed by *Bacillus subtilis*, which showed an MIC of 375 µg/mL. *Escherichia coli*, a Gram-negative organism, exhibited reduced susceptibility with an MIC of 500 µg/mL, while *Pseudomonas aeruginosa* showed the highest resistance with an MIC of 500 µg/mL. These results support the formulation’s preferential action against Gram-positive bacteria, likely due to differences in cell wall structure and permeability. Although the MIC values were higher than conventional antibiotics, Melissa oil’s broad-spectrum antimicrobial activity and favorable safety profile make M-NG suitable for topical use. The formulation may be particularly beneficial in managing localized skin infections, such as acne, burns, and minor wounds, where both bacterial colonization and inflammation contribute to delayed healing. The dual antimicrobial and anti-inflammatory properties of Melissa oil enhance the therapeutic utility of M-NG in dermatological care.

### 2.13. Examination of Dermal Effects in the Acute Irritation Study

The acute skin irritation potential of the Melissa Nanoemulgel (M-NG) formulation was evaluated following OECD Guideline 404, using three groups of healthy male Wistar rats (*n* = 3 per group). The study aimed to evaluate any observable skin reactions following a single dose application of M-NG. The treated area was observed at 1, 24, 48, and 72 h post-application, and the severity of dermal responses was scored using a standard irritation scale (Table 5). At the 1-h mark, the M-NG group showed slight erythema (median score: 0.5 ± 0.5), which completely resolved by the 24-h observation point. No edema or other visible irritation symptoms were recorded at any time point. In comparison, the positive control group (treated with Formalin) exhibited notable erythema and mild edema (median score: 2.5 ± 0.5 at 1 h), with signs of irritation persisting through 72 h, although gradually diminishing in intensity. The negative control group (treated with Blank Gel) displayed no erythema or edema throughout the observation period. Statistical analysis using the Kruskal–Wallis test demonstrated a significant difference (*p* < 0.05) between the test groups at all time points, particularly between the M-NG and Formalin groups. These results confirm that M-NG causes negligible dermal irritation, comparable to the blank control, and is significantly less irritating than the positive control. This supports its safety and compatibility for topical application, indicating potential for dermatological use without risk of adverse local skin reactions.

### 2.14. In Vivo Assessment of Anti-Inflammatory Activity

**Figure 10:**
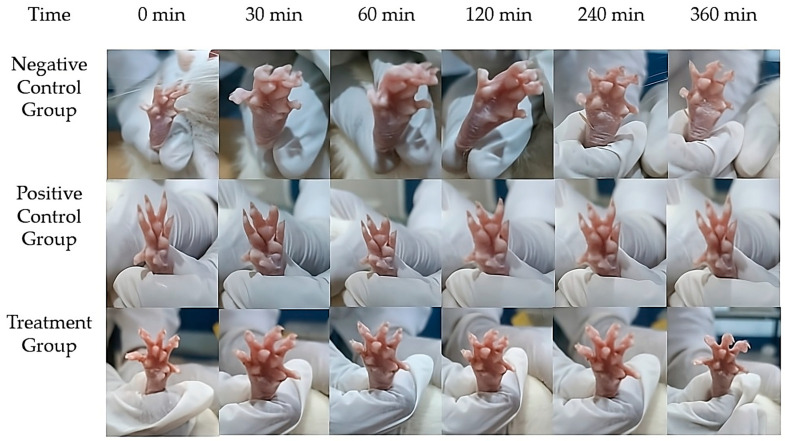
Time-course images showing anti-inflammatory effects in carrageenan-induced paw edema in rats. The Treatment Group (Melissa Nanoemulgel) showed faster and greater reduction in swelling compared to the Positive Control (Diclofenac gel) and Negative Control (blank gel), with complete resolution by 360 min.

The anti-inflammatory activity of the Melissa Nanoemulgel (M-NG) was evaluated using the carrageenan-induced paw edema model in Wistar rats. The study compared three groups: a test group treated with M-NG, a standard group treated with a marketed Diclofenac gel (2.5% *w*/*w*), and a control group receiving blank nanoemulgel (Figure 10). Paw swelling was induced via subplantar injection of carrageenan, and the changes in paw volume were measured at 0, 30, 60, 120, 240, and 360 min using a plethysmometer (Table 6). The percent inhibition of edema was calculated at each time point. At 30 min post-induction, the M-NG group showed a reduction in paw volume to 0.25 ± 0.006 mL, compared to 0.32 ± 0.005 mL in the standard group and 0.42 ± 0.007 mL in the control. The edema inhibition was 40.5% for M-NG and 23.8% for the standard group. Statistically significant differences were observed between the M-NG and control groups (*p* = 0.029). At 60 min, M-NG exhibited 60.3% inhibition (volume: 0.17 ± 0.004 mL) compared to 33.2% in the standard group (0.28 ± 0.005 mL), with a highly significant difference (*p* = 0.008). At 120 min, M-NG further reduced paw volume to 0.07 ± 0.003 mL (81.6% inhibition), while the standard maintained a moderate effect (0.19 ± 0.004 mL, 52.6% inhibition). Although the M-NG group consistently outperformed the standard, the difference did not reach statistical significance at this time point (*p* = 0.076). At 240 min, inflammation in the M-NG group was nearly eliminated (0.01 ± 0.001 mL, 96.9% inhibition), compared to the standard group (0.05 ± 0.001 mL, 86.5% inhibition). At 360 min, complete resolution of edema was observed in both M-NG and standard groups (100% inhibition), while the control group still showed residual swelling (0.33 ± 0.002 mL). These findings confirm the superior anti-inflammatory potential of Melissa Nanoemulgel over the standard treatment. The formulation demonstrated rapid onset, sustained effect, and complete resolution of inflammation without adverse effects, establishing its promise for topical management of inflammatory skin conditions.

## 3. Conclusions

We used a design (QbD) strategy, focusing on Tween 80 concentration and homogenization time as key formulation variables. FTIR spectroscopy confirmed the presence of functionally active bio-compounds, supporting the medicinal potential of *Melissa* oil. The optimized formulation exhibited desirable physicochemical properties, including a droplet size of 127.31 nm, a polydispersity index (PDI) of 17.7%, a zeta potential of −25.0 mV, and light transmittance of 88.3%, indicating that the nanoemulsion was stable, homogeneous, and appropriate for transdermal delivery. Drug release studies conducted in vitro demonstrated that the release pattern best fit the Higuchi model (R^2^ = 0.990), indicating diffusion-dominated behavior. Additionally, results from the Korsmeyer–Peppas model (*n* = 0.88) suggested that the release followed a non-Fickian, or anomalous, transport mechanism. Antibacterial evaluations demonstrated effective suppression of microbial growth across four bacterial strains—*Staphylococcus aureus*, *Escherichia coli*, *Bacillus subtilis*, and *Pseudomonas aeruginosa*—with Gram-positive strains showing greater susceptibility. In particular, *S. aureus* exhibited the lowest MIC, indicating the highest sensitivity to the formulation. The anti-inflammatory efficacy of the formulation was further supported by in vivo studies, which showed a statistically significant reduction in paw edema (*p* = 0.005 at 60 min) and nearly full resolution by 240 min. This study followed CPCSEA ethical guidelines and incorporated the 3Rs principle (Replacement, Reduction, and Refinement) to ensure humane animal handling. While only male Wistar rats were used to reduce hormonal variability, this is recognized as a study limitation, and future work should include both sexes to better assess therapeutic generalizability. Overall, these findings support the potential of *Melissa officinalis* oil-based nanoemulgel as a novel transdermal delivery platform with dual anti-inflammatory and antimicrobial properties, suitable for treating skin-related infections and inflammatory conditions.

## 4. Materials and Methods

### 4.1. Materials

Melissa essential oil was obtained from Avi Naturals, Delhi, India. Additional components of the formulation, such as Carbopol 934, benzyl alcohol, and sodium hydroxide (NaOH), were acquired from Loba Chemie Pvt. Ltd., Mumbai, India. The bacterial strains used for microbiological testing included *Staphylococcus aureus* (MTCC 737) and *Escherichia coli* (MTCC 1035), both of which were provided by the Microbial Type Culture Collection and Gene Bank (MTCC), Chandigarh, India.

### 4.2. Surfactant Screening and FT-IR Analysis of Melissa Oil

A structured screening process was conducted to determine the most effective surfactant for developing oil-in-water (O/W) nanoemulsions containing Melissa oil. Various surfactant types were tested, including polysorbates (Tween 80 and Tween 20), sorbitan esters (Span 80 and Span 20), and polyethylene glycol 400 (PEG 400). Each formulation included 5% *w*/*v* of Melissa oil, while the concentration of surfactants was varied between 1% and 20% *w*/*v*. The evaluation involved parameters such as droplet size, emulsion stability, and clarity to identify the optimal surfactant. Tween 80 emerged as the most suitable candidate, offering the best emulsification capacity, consistent droplet formation, and formulation stability. Consequently, Tween 80 was selected for use in the final formulation [11].

Fourier-transform infrared (FT-IR) spectroscopy of Melissa oil was conducted using an attenuated total reflectance (ATR) accessory (Lab India-Bruker, Mumbai, India). A small amount of oil was placed directly on the ATR crystal to ensure adequate contact. The spectra were recorded over a range of 4000 to 400 cm^−1^ with a resolution of 4 cm^−1^, averaging 32 scans to improve precision. Background correction was performed before data acquisition. The obtained spectrum was then examined to identify the characteristic functional groups present in Melissa oil.

### 4.3. Development and Optimization of Melissa Nanoemulsion

Preliminary formulation work identified that a 5% *w*/*v* concentration of *Melissa* essential oil was optimal for developing the nanoemulsion. The nanoemulsion was formulated through an oil-in-water (O/W) emulsification method. During this process, the aqueous phase—consisting of distilled water (89–93% *w*/*v*) and Tween 80 (2–6% *w*/*v*)—was incrementally added to the oil phase while stirring continuously at 40 °C using a Remi magnetic stirrer, India for 1 h to achieve homogeneous mixing. The resulting coarse emulsion was subsequently homogenized at a high shear speed (1400 rpm) for 10 to 30 min to produce a stable nanoemulsion system [12]. The two independent variables chosen for optimization Tween 80 concentration (X_1_) and homogenization time (X_2_) were selected based on their critical influence on emulsion stability, droplet size, and surfactant efficiency, as observed in preliminary trials and supported by prior studies on nanoemulsion systems. Tween 80 was selected for its proven emulsifying capability and biocompatibility, while homogenization time was varied to modulate shear energy and droplet breakup.

To systematically optimize the formulation, a Central Composite Design (CCD) approach with two independent variables was implemented using Design Expert Software (Version 13, Stat-Ease Inc., Minneapolis, MN, USA). The variables investigated were Tween 80 concentration (2%, 4%, and 6%) and homogenization time (10, 20, and 30 min), each tested at five levels (including axial points). The measured responses included droplet size (Y_1_), which was aimed to be minimized, and zeta potential (Y_2_), which was maintained within an acceptable stability window. Thirteen experimental formulations were prepared, including replicated center points and axial points to ensure sufficient prediction accuracy and model robustness. A summary of the CCD matrix, including actual and coded values of the formulation variables, is presented in Table 2. Analysis of variance (ANOVA) was carried out at a 95% confidence interval, applying a statistical significance threshold of *p* ≤ 0.05. The formulation demonstrating optimal results, i.e., minimum droplet size and desirable zeta potential—was selected for detailed evaluation and characterization.

### 4.4. Assessment of Entrapment Efficiency

The entrapment efficiency (EE%) of the Melissa oil nanoemulsion (M-NE) was determined using a modified UV-visible spectrophotometric procedure, adapted from previously reported protocols [13]. A standard calibration curve was constructed at the maximum absorbance wavelength (λmax = 274 nm), with concentrations ranging from 1 to 15 µg/mL. The method showed excellent linearity with a regression coefficient (R^2^) of 0.998, confirming its quantitative accuracy. The sensitivity of the method was validated by calculating the limit of detection (LOD = 0.46 µg/mL) and limit of quantification (LOQ = 1.44 µg/mL), based on standard deviation and slope analysis [14]. For the EE assay, 1 mL of the nanoemulsion was placed into a centrifugal ultrafiltration tube (Amicon^®^ India, 10 kDa MWCO) and centrifuged at 10,000 rpm for 30 min to separate the unentrapped (free) oil. The clear filtrate was collected, diluted in a 1:1 (*v*/*v*) ethanol–distilled water mixture, and analyzed at 274 nm using a UV–Vis spectrophotometer. Blank nanoemulsion (without oil) was used as the baseline to eliminate background interference. All experiments were performed in triplicate, and the EE% was calculated using the following equation:

$$EE\%=\left(\frac{W_{total}-W_{free}}{W_{total}}\right)\times 100$$

where W_total_ refers to the total amount of Melissa oil initially added to the nanoemulsion formulation, and W_free_ represents the amount of unentrapped (free) oil present in the filtrate following ultrafiltration. The difference between these values reflects the encapsulated portion, allowing accurate calculation of the entrapment efficiency.

### 4.5. Physicochemical Characterization and Stability Assessment

The Melissa oil nanoemulsions were characterized for their physicochemical properties, such as particle size, polydispersity index (PDI), and zeta potential, employing the Malvern Zetasizer Nano ZS 90, India with photon correlation spectroscopy. Particle size and PDI measurements were carried out at 25 °C using disposable polystyrene cuvettes, while zeta potential was analyzed using omega cuvettes, India. [15]. Stability of the nanoemulsion formulations was assessed through stress testing involving thermal and freeze–thaw cycles. For thermal stress, samples underwent alternating heating and cooling cycles between 40 °C and 48 °C, each cycle lasting 48 h and repeated three times. Following each cycle, the samples were checked for any signs of phase separation or instability. For freeze–thaw testing, the emulsions were frozen at −20 °C and thawed at room temperature (25 °C) across three cycles. After completion, centrifugation was performed to evaluate physical stability and detect any formulation breakdown [16].

### 4.6. Scanning Electron Microscopy Analysis

Scanning Electron Microscopy (SEM) was employed to investigate the microstructural morphology of the lyophilized Melissa nanoemulsion (M-NE) to understand particle surface characteristics and distribution uniformity. A small amount of the freeze-dried nanoemulsion was carefully mounted onto an aluminium stub using conductive carbon adhesive tape. The sample was then coated with a thin layer of gold (~10 nm) using a sputter coater under a high-vacuum chamber to prevent charging during imaging. SEM analysis was carried out on a ZEISS scanning electron microscope, India under high-vacuum mode using secondary electron imaging (Signal A = SE2), at an accelerating voltage of 10.00 kV and a working distance of 10.45 mm. Images were acquired at different magnifications with scale bars of 20 µm and 10 µm to allow both surface and morphological evaluations [17].

### 4.7. Preparation of Melissa Oil-Loaded Nanoemulgel

The nanoemulgel comprising Melissa oil (M-NG) was developed using Carbopol 940 as the gelling matrix at concentrations varying between 0.5% and 2% *w*/*w*. The gelling agent was slowly incorporated into the pre-formulated Melissa nanoemulsion with continuous agitation to ensure homogeneity. To enhance preservation and maintain product stability, benzyl alcohol was included, and the pH was finely tuned with 1 M sodium hydroxide to ensure suitable skin tolerance. The final formulation was evaluated for physical properties including appearance, clarity, homogeneity, foaming tendency, and leakage. A Thumb Test was used to examine spreadability and adhesion. Spreadability was determined via the glass slide technique, and viscosity was evaluated using a Brookfield viscometer, India (ULA S00 spindle, 4 rpm, torque at level 10) [18]. pH stability was monitored to evaluate compatibility and long-term storage potential with skin.

### 4.8. Characterization of Melissa Nanoemulgel

The prepared nanoemulgel was characterized for its macroscopic appearance, rheological behavior, and spreadability to assess its suitability for topical application. Macroscopic evaluation involved visual inspection for color, consistency, clarity, homogeneity, and signs of phase separation or syneresis. The formulation exhibited a smooth, uniform texture and was assessed by manually rubbing a small amount between two clean glass slides. Rheological behavior was evaluated using a Brookfield viscometer (Model LV-DV-II+, Brookfield Engineering Labs, Massachusetts, USA) fitted with spindle number 64 at 25 ± 1 °C. Viscosity measurements were recorded at spindle speeds ranging from 10 to 100 rpm. For each speed, readings were performed in triplicate (*n* = 3) and the mean viscosity ± standard deviation (SD) was calculated [19]. The results were graphically presented as a viscosity vs. spindle speed curve with error bars (±SD), and capped ends were used to represent variability across replicates. This ensured clear visual interpretation of the formulation’s flow behavior. Spreadability was determined using the glass slide method, where 0.5 g of gel was placed between two glass slides and compressed with a 500 g weight for 5 min. The diameter of the circular spread area was measured in centimeters. While spreadability (S) can be calculated using the formula

$$S=\frac{m\times d}{t}$$

where *m* is the applied weight (g), *d* is the diameter of spread (cm), and *t* is time (s), in this study the diameter was reported directly for simplicity. All tests were conducted in triplicate to ensure reproducibility and accuracy.

### 4.9. Differential Scanning Calorimetry Analysis

Differential scanning calorimetry (DSC) was conducted to investigate the thermal behavior of *Melissa officinalis* oil and the developed nanoemulgel formulation. The analysis was carried out using a DSC Q20 (TA Instruments, Delaware, USA) equipped with a refrigerated cooling system and operated with Universal Analysis software (v4.5A). Approximately 10.0 ± 0.2 mg of each sample (Melissa oil and Melissa nanoemulgel) was accurately weighed and sealed in standard aluminium pans, with an empty sealed pan used as a reference. The thermal scans were performed in a nitrogen atmosphere (flow rate: 50 mL/min) to prevent oxidative degradation, and the temperature was ramped from 25 °C to 300 °C at a heating rate of 10 °C/min [20].

### 4.10. Analysis of In Vitro Drug Diffusion and Kinetic Modeling

The release profile of the Melissa nanoemulgel (M-NG) was analyzed using a modified Franz diffusion cell system [21]. A cellophane membrane with 12 kDa molecular cut-off was pre-soaked in a 1:1 ethanol–water mixture overnight for hydration. About 1 g of M-NG was positioned in the donor compartment. At regular time intervals, samples were collected from the receptor chamber, replacing it with an equal amount of fresh medium each time to ensure sink conditions. The system was maintained at 32 °C with continuous stirring at 200 rpm throughout the experiment [22]. The samples were diluted and analyzed using UV-spectrophotometry at the maximum absorbance of Melissa oil. A previously constructed standard curve was used for quantification. A placebo formulation (without Melissa oil) served as a blank to prevent any interference from excipients. Additionally, the release pattern of a nanoemulsion formulation without Carbopol (M-NE), free Melissa oil solution, and a conventional hydrogel with Melissa oil in 1% Carbopol 940 were also evaluated for comparison. The findings were reported as mean ± SD from three replicates. The release data were fitted into different kinetic models such as zero-order, first-order, Higuchi, Hixson–Crowell, and Korsmeyer–Peppas to determine the release mechanism [23]. The model with the highest R^2^ value was used to determine the most accurate release mechanism.

### 4.11. Accelerated Stability Study

To assess the physical stability of the optimized Melissa oil nanoemulgel (M-NG) and predict its long-term behavior, an accelerated stability study was conducted following ICH guidelines (Q1A(R2)) [24]. The nanoemulgel was stored in tightly sealed, opaque HDPE containers and kept in a stability chamber at 40 ± 2 °C and 75 ± 5% relative humidity (RH) for 30 days. At predetermined intervals (Day 0, Day 15, and Day 30), samples were withdrawn and analyzed for changes in macroscopic appearance (color, homogeneity, phase separation, syneresis), texture, viscosity, spreadability, and centrifugation stability. Visual and tactile inspections were conducted to observe any signs of separation or deterioration. Viscosity was measured at 25 ± 1 °C using a Brookfield viscometer, India (Model LV-DV-II+, spindle 64) at 10 rpm, while spreadability was determined by placing 0.5 g of the sample between two glass slides under a 500 g weight for 5 min, and the resulting diameter of spread was measured in centimeters. Phase separation was further evaluated by centrifuging samples at 3000 rpm for 30 min. All tests were conducted in triplicate to ensure consistency and reliability of the observations.

### 4.12. Assessment of Antibacterial Potential of M-NG Formulation

The antimicrobial efficacy of the Melissa nanoemulgel (M-NG) was assessed against four bacterial strains, *Staphylococcus aureus* (MTCC 737), *Escherichia coli* (MTCC 1035), Pseudomonas aeruginosa (MTCC 1688), and Bacillus subtilis (MTCC 441), using the broth microdilution assay, conducted in accordance with the CLSI (Clinical and Laboratory Standards Institute) guidelines [25]. A series of two-fold serial dilutions of M-NG were prepared in Mueller–Hinton Broth (MHB) using a 96-well sterile microtiter plate, with concentrations ranging from 500 µg/mL to 1.95 µg/mL. Fresh cultures of the test organisms were grown on Nutrient Agar and adjusted to a 0.5 McFarland turbidity standard (~1.5 × 10^8^ CFU/mL) by suspending them in sterile saline. This suspension was then diluted to achieve a final inoculum of approximately 0.5 × 10^5^ CFU/mL per well, and 100 µL of the bacterial suspension was added into each well. The microplates were incubated at 37 °C for 24 h under sterile conditions. Bacterial growth was evaluated spectrophotometrically at 600 nm (OD600). The Minimum Inhibitory Concentration (MIC) was defined as the lowest concentration of M-NG showing no visible growth and OD ≤ 0.1. To validate the assay, sterility control (MHB only), growth control (bacteria without test formulation), and positive control (ciprofloxacin 2 µg/mL) were included. All experiments were performed in triplicate, and results were expressed as mean ± standard deviation (SD).

### 4.13. Preclinical Evaluation Using Animal Models

The in vivo study was conducted using male Wistar rats (body weight: 250 ± 10 g). Only male Wistar rats were used in this study to reduce hormonal variability; however, this represents a limitation, and future studies should include both sexes to better assess the formulation’s efficacy across biological differences. The animals were housed in a controlled environment with temperature maintained at 25 ± 2 °C, a 12-h light/dark cycle, and unrestricted access to pellet feed and clean drinking water [26]. All experiments adhered to ethical norms specified by the Committee for the Purpose of Control and Supervision of Experiments on Animals (CPCSEA) [27] and were carried out only after receiving approval from the Institutional Animal Ethics Committee (IAEC) under protocol number 07/IAEC/CLPT/2023-24 (Reg. No: 1048/PO/Re/S/07/CPCSEA). The study design ensured animal welfare and ethical compliance throughout the experimental timeline.

### 4.14. Evaluation of Skin Irritation Following Topical Application

The potential for skin irritation by the Melissa nanoemulgel (M-NG) was assessed following the OECD guideline 404 [28] using healthy male Wistar rats. An amount of 0.5 g of M-NG was applied on a 6 cm^2^ shaved skin area and covered with a semi-occlusive dressing for 4 h. As a control, 0.8% formalin solution served as a positive control, while a blank gel served as the negative control. Following exposure, the area was cleaned with warm water, and observations were made for skin reactions such as redness (erythema) and swelling (edema) at 1, 24, 48, and 72 h post-treatment.

### 4.15. Investigation of Anti-Inflammatory Efficacy in Animal Models

The anti-inflammatory activity of the Melissa oil-loaded nanoemulgel (M-NG) formulation was evaluated using the carrageenan-induced paw edema model in male Wistar rats. A total of 18 healthy rats (*n* = 6 per group; weight range: 200–300 g) were randomly divided into three groups: Group 1 received blank nanoemulgel (negative control), Group 2 received Dicloran^®^ gel containing 2.5% diclofenac sodium (positive control), and Group 3 was treated with the M-NG formulation containing 5% *w*/*w Melissa officinalis* oil. The animals were housed under standard laboratory conditions (25 ± 2 °C, 12 h light/dark cycle) with ad libitum access to food and water. The study protocol was approved by the Institutional Animal Ethics Committee (IAEC) under approval number [insert IAEC number], and all procedures were conducted in accordance with CPCSEA guidelines. To ensure ethical transparency, the study followed the principles of the 3Rs—Replacement, Reduction, and Refinement. The number of animals used was the minimum required to achieve statistical significance. Efforts were made to minimize pain and distress by providing environmental enrichment, proper acclimatization before the experiment, and ensuring all observations were conducted by a blinded observer to reduce handling stress.

To induce inflammation, 0.1 mL of 1% *w*/*v* carrageenan solution was injected into the subplantar region of the right hind paw of each animal. Immediately after the injection, 0.5 g of the assigned formulation was applied topically over the inflamed paw and gently massaged to ensure even absorption. The treatment was applied once. Paw volume was measured at 0 (baseline), 30, 60, 120, 240, and 360 min post-carrageenan injection using a digital vernier caliper by an observer blinded to the treatment groups.

The percentage change in paw volume was calculated using the following formula:

$$\%\;Change\;in\;Hind\;Paw\;Volume=\left(\frac{Mean\;C_{n}-Mean\;C_{i}}{Mean\;C_{i}}\right)\times 100$$

where Mean Cₙ is the average paw volume at each time point, and Mean Cᵢ is the baseline paw volume prior to carrageenan injection. To determine the anti-inflammatory efficacy, the percentage inhibition of paw edema was calculated by comparing treated groups to the negative control. All values are expressed as mean ± standard deviation (SD). Statistical significance was determined using one-way ANOVA followed by Tukey’s post hoc test, with *p* < 0.05 considered statistically significant [29].

### 4.16. Statistical Analysis and Interpretation

Data obtained from the experiments were statistically analyzed using Student’s *t*-test for comparisons between two groups and one-way analysis of variance (ANOVA) followed by the Tukey–Kramer post hoc test for multiple group comparisons. Results from in vitro evaluations were reported as mean ± standard deviation (SD), whereas in vivo data were presented as mean ± standard error of the mean (SEM). A *p*-value of less than 0.05 was considered indicative of statistical significance, thereby confirming the robustness and reproducibility of the experimental outcomes.

## Figures and Tables

**Table 1:** Characteristic FTIR Bands of Melissa Oil and Corresponding Functional Groups.

Observed Wavenumber (cm^−1^)	Assigned Functional Group	Typical Wavenumber Range (cm^−1^)	Presumed Compound Class
3821.54, 3740.35	O–H stretch	3200–3700	Alcohols, Phenols
3498.79	H-bonded O–H stretch	3200–3600	Phenolics, Hydroxyl groups
2921.97, 2863.12, 2734.83	C–H stretch	2800–3000	Alkanes (saturated hydrocarbons)
1730.00, 1672.26	C=O stretch	1650–1750	Esters, Ketones
1510.91, 1445.89, 1378.71	C=C stretch, CH bend	1350–1600	Aromatics, Alkanes, Alkenes
1237.02, 1180.75, 1113.96, 1025.96	C–O stretch	1000–1300	Alcohols, Ethers
987.69, 925.59	=C–H bending	800–1000	Terpenes, Aromatics
835.55, 750.74, 693.16	C–H out-of-plane bend	650–900	Aromatic or cyclic compounds
593.15, 533.13, 459.41	Fingerprint region	<600	Specific spectral features

**Table 2:** Experimental Design Matrix with actual and coded levels of variables, and Observed Particle Size and Zeta Potential.

Formulations	X_1_ (Coded Level)	X_2_ (Coded Level)	Tween 80 (%) X_1_	Homogenization Time (min) X_2_	Particle Size (nm)	Zetapotential
1	0	0	4	20	147.7	−25
2	0	0	4	20	129.5	−24.9
3	−	−	2	10	201.1	−12.8
4	0	0	4	20	132.9	−25.4
5	0	A	4	34.1421	110.98	−25
6	0	0	4	20	140.8	−24.1
7	+	+	6	30	119.1	−28.7
8	−	+	2	30	150.6	−21.8
9	a	0	1.17157	20	160.28	−15.9
10	+	−	6	10	181.34	−22.8
11	0	0	4	20	139.67	−23.7
12	A	0	6.82843	20	132.89	−25.1
13	0	a	4	5.85786	173.52	−15.8

**Table 3:** Statistical Analysis of Model Parameters Using ANOVA.

Factor	df	Overall Variability	Mean Square	F-Statistic	*p*-Value	Statistical Significance
Model	5	6564.29	1312.86	12.47	0.0021	Significant
Error	7	736.71	105.24	–	–	–
Total	12	7301	–	–	–	–
Intercept	–	–	143.92	28.72	<0.0001	Highly significant
Tween 80	1	–	−23.56	−4.11	0.0047	Highly significant
Homogenization Time	1	–	−27.89	−5.24	0.0022	Highly significant
Tween 80 × Homogenization Time	1	–	1.89	0.34	0.7442	Not significant
Tween 80 × Tween 80	1	–	9.63	2.71	0.0481	Marginally significant
Homogenization Time × Time	1	–	5.74	1.28	0.2417	Not significant
R^2^	–	0.8992	–	–	–	Indicates strong model fit
Adjusted R^2^	–	0.8465	–	–	–	Adjusted for predictors

**Table 4:** Comparative Drug Release Kinetic Models for M-NG Formulation.

Kinetic Model	Equation	R^2^ Value	Interpretation
Zero-order	$Q_{t}=Q_{0}+k_{0}t$	0.825	Constant release rate (less suited here)
First-order	$logQ_{t}=logQ_{0}-\frac{k_{1}t}{2.303}$	0.841	Concentration-dependent release
Higuchi	$Q_{t}=k_{H}\;\sqrt{t}$	0.900	Best fit: diffusion-controlled release
Korsmeyer– Peppas	$\frac{M_{t}}{M_{\infty }}=kt^{n}$	0.895	Non-Fickian (anomalous) transport (*n* = 0.88)
Hixson–Crowell *(optional)*	${Q_{0}}^{1/3}-{Q_{t}}^{1/3}=kt$	0.810	Suggests erosion/dissolution (less relevant here)

Note: Here, Q_t_ represents the amount of drug released at time t, and Q_0_ is the initial drug content. M_t_/M_∞_ denotes the fraction of drug released at time t. The terms k_0_, k_1_, k_H_, and k are the respective rate constants for the zero-order, first-order, Higuchi, and Korsmeyer–Peppas models. The exponent *n* in the Korsmeyer–Peppas equation characterizes the release mechanism.

**Table 5:** Acute Dermal Irritation Scores of different groups.

Time Point	Melissa Nanoemulgel ^1^	Positive Control (Formalin) ^1^	Negative Control (Blank Gel) ^1^	*p*-Value ^2^
1 h	0.5 ± 0.5	2.5 ± 0.5	0.0 ± 0.0	0.0274
24 h	0.0 ± 0.0	2.0 ± 0.5	0.0 ± 0.0	0.0196
48 h	0.0 ± 0.0	1.5 ± 0.5	0.0 ± 0.0	0.0221
72 h	0.0 ± 0.0	1.0 ± 0.0	0.0 ± 0.0	0.0303

^1^ *n* = 3 (expressed as median ± interquartile range). ^2^ *p* < 0.05, indicating a statistically significant difference between groups (Kruskal–Wallis test).

**Table 6:** In Vivo Evaluation of Anti-Inflammatory Activity: Edema Reduction and Statistical Analysis.

Time (min)	Negative Control Group (mL) ^1^	Positive Control Group (mL) ^1^	Treatment Group (mL) ^1^	Inhibition of Edema (%)	Group Comparisons	Mean Difference (mL)	*p*-Value ^4^
0	0.00 ± 0.00	0.00 ± 0.00	0.00 ± 0.00	-	-	-	-
30	0.42 ± 0.007	0.32 ± 0.005	0.25 ± 0.006	23.8 ^2^/ 40.5 ^3^	Negative Control vs. Standard	−0.17	0.029
60	0.41 ± 0.006	0.28 ± 0.005	0.17 ± 0.004	33.2 ^2^/ 60.3 ^3^	Negative Control vs. Treatment	−0.24	0.008
120	0.39 ± 0.005	0.19 ± 0.004	0.07 ± 0.003	52.6 ^2^/ 81.6 ^3^	Standard vs. Treatment	−0.12	0.076
240	0.36 ± 0.003	0.05 ± 0.001	0.01 ± 0.001	86.5 ^2^/ 96.9 ^3^	-	-	-
360	0.33 ± 0.002	0.00 ± 0.00	0.00 ± 0.00	100 ^2,3^	-	-	-

^1^ Mean ± Standard Error of the Mean (*n* = 6). ^2^ Percentage inhibition calculated in comparison with the Positive Control Group (Standard, S). ^3^ Percentage inhibition calculated relative to the Treatment Group (T). ^4^ A *p*-value less than 0.05 was considered statistically significant.

## Data Availability

The data generated in this study can be requested from the corresponding author.

## Abbreviations

The following abbreviations are used in this manuscript:

QbD	Quality by Design
NE	Nanoemulsion
NEs	Nanoemulsions
M-NE	Melissa Nanoemulsion
n-EnM	Nanoemulsion in Matrix
n-EnM Hydrogel	Nanoemulsion in Hydrogel
M-NG	Meliss Nanoemulgel
CCD	Central Composite Design
ANOVA	Analysis of Variance
PDI	Polydispersity Index
CI	Confidence Interval
EE	Entrapment Efficiency
LOD	Limit of Detection
LOQ	Limit of Quantification
SEM	Scanning Electron Microscopy
DSC	Differential Scanning Calorimetry
UV	Ultraviolet
O/W	Oil in Water
MHB	Mueller–Hinton Broth
MIC	Minimum Inhibitory Concentration
CLSI	Clinical and Laboratory Standards Institute
CFU	Colony-Forming Unit
IAEC	Institutional Animal Ethics Committee
